# Gender Is a Significant Prognostic Factor for Upper Tract Urothelial Carcinoma: A Large Hospital-Based Cancer Registry Study in an Endemic Area

**DOI:** 10.3389/fonc.2019.00157

**Published:** 2019-03-21

**Authors:** Chun-Chieh Huang, Yu-Li Su, Hao-Lun Luo, Yen-Ta Chen, Terence T. Sio, Hsuan-Chih Hsu, Chia-Hsuan Lai

**Affiliations:** ^1^Department of Radiation Oncology, Kaohsiung Chang Gung Memorial Hospital, Chang Gung University College of Medicine, Kaohsiung, Taiwan; ^2^Graduate Institute of Clinical Medical Sciences, College of Medicine, Chang Gung University, Taoyuan, Taiwan; ^3^Division of Hematology-Oncology, Department of Internal Medicine, Kaohsiung Chang Gung Memorial Hospital, Chang Gung University College of Medicine, Kaohsiung, Taiwan; ^4^Department of Urology, Kaohsiung Chang Gung Memorial Hospital, Chang Gung University College of Medicine, Kaohsiung, Taiwan; ^5^Department of Radiation Oncology, Mayo Clinic Hospital, Phoenix, AZ, United States; ^6^Department of Radiation Oncology, Chiayi Chang Gung Memorial Hospital, Chiayi, Taiwan

**Keywords:** upper urinary tract, urothelial carcinoma, gender, nephroureterectomy, renal pelvis, ureter

## Abstract

**Introduction:** Our hospital is a tertiary medical center located in southern Taiwan, which is an endemic area for upper tract urothelial carcinoma (UTUC) cases. Using a large registry-based surgical database, we examined our cohort of patients with UTUC, and evaluated the treatment outcome and gender-specific differences in this population.

**Methods:** A total of 506 patients with localized UTUC undergoing nephroureterectomy from 2004 to 2013 were enrolled. The patient, tumor, and treatment-related characteristics were prospectively recorded by the registry. Overall (OS) and cancer-specific (CSS) survival outcomes were evaluated as well. Gender differences as related to clinical and pathological factors were examined by chi-square testing. Univariate and multivariate Cox regression analyses were applied.

**Results:** There were more female patients (57.9%) in this population. The median follow-up was 6.9 years for living patients. The actuarial 5-year OS and CSS rates were 69.4 and 84.9%, respectively. Being female, aged <70 years, and early T-stage were statistically significantly associated with better OS and CSS by multivariate analyses. The 5-year CSS rates for females vs. males were 89.6 and 78.5%, respectively (*P* < 0.005). A subgroup analysis suggested that better survival outcomes for females only existed in the stage 0a/0is/I (non-muscle-invasive), but not in the advanced stage.

**Conclusions:** In an endemic area, females were more likely diagnosed with UTUC, but had significantly improved OS and CSS compared to their male counterparts, which were mostly driven by the non-muscle-invasive cases. Future research should focus on better understanding the epidemiologic risk-factor profile and pathophysiologic differences based on gender.

## Introduction

Upper tract urothelial carcinoma (UTUC) is uncommon and only accounts for 5–10% of urothelial carcinomas worldwide (1, 2). Incidence of UTUC in the United States has been reported as low as 2.06 cases per 100,000 person-years from a study based on the database of Surveillance, Epidemiology and End Results (SEER) (3). Radical nephroureterectomy is the standard treatment for most patients with localized UTUC.

Several large multicenter series have reported the gender-associated outcome in UTUC. One study (4) enrolling 1,363 patients from 12 centers reported twice as many males as females (67.6 vs. 32.4%), and the disease-specific survival was not significantly different between males and females. Another study (5) with 754 patients from 9 centers also reported more males than females (68.4 vs. 31.6%), and the gender difference did not influence cancer-specific survival (CSS). Another SEER-based study (6) including 4,850 patients also reported more male cases compared to females (59.9 vs. 40.1%). A higher cancer-specific mortality rate was found in females, but not significant by multivariate analysis. However, none of these studies above was majorly based in Asia (4–6).

Over the years, in our daily practice of diagnosis, treatment, and follow-up for patients with UTUC, we notice a different gender distribution and gender-associated outcome as compared with the reports from other population series globally. Our hospital is a large tertiary referral center located in southern Taiwan, which is an endemic area for UTUC. Our unique population also has a high prevalence of herbal medicine use as a dietary supplement. Some herbal medicine contains aristolochic acid, which is an urothelial carcinogen and might contribute to higher prevalence of UTUC in our area (7, 8). According to the Taiwan Cancer Registry Annual Report, the crude incidence rate of urothelial carcinoma in urologic malignancy excluding bladder cancer is as high as 5.25 males and 6.77 females per 100,000 person-years in 2015 (9), or 5.8 times higher than the American population.

We are reporting a large series from Taiwan, an endemic area of UTUC, to complement the previous series that have been reported, and highlighted the unique gender differences and patterns that are only seen in our series. Additionally, we are also reporting a modern series, as compared to previous clinical reports which had smaller number of cases or with patient cases from earlier decades. Using our hospital's cancer registry data, we analyzed and evaluated the gender difference and prognostic factors in patients with UTUC after radical nephroureterectomy. All cases were treated with curative intent upfront.

## Materials and Methods

### Study Population and Variables

This study was approved by the Institutional Review Board (IRB) at our institution (No. 201601404B0). The need for individual informed consent from each patient was waived by the IRB as only cancer registry data which were prospectively collected and posed minimal risk to the patients who were already diagnosed and treated.

The study was initiated in November 2016. A total of 1,573 patient cases diagnosed with renal pelvis cancer or ureter cancer (ICD-O-3 C65.9 or C66.9) between 1995 and 2014 were acquired from our hospital's cancer registry database which was maintained prospectively. The following patients were sequentially excluded: 537 patients either with previous cancer history or registered twice due to synchronous renal pelvis cancer or ureter cancer; 5 with histology of non-urothelial carcinoma; 67 with distant metastases at diagnosis; 175 without nephroureterectomy; 206 with unknown T-stage; 12 diagnosed before January 1, 2004; and 65 diagnosed after December 31, 2013. As a result, 506 remaining patients diagnosed with *de novo* UTUC between 2004 and 2013 were enrolled for analysis. All of them received nephroureterectomy and had non-metastatic disease at initial diagnosis. The reason for the exclusion of data before 2004 was that our hospital began to regularly register every new case of UTUC into our cancer registry database starting in 2002. With 2 years' practice, we assumed that the quality of registration would be more stable and ever reliable as a result. We also excluded the new cases after 2013 so that our patients would have at least 2 years of minimum follow-up in our cohort.

The collected registry data for final analysis included gender, age at diagnosis, tumor location and laterality, synchronous status of UTUC, tumor grade and architecture, pathological T-stage, pathological N-stage, stage group according to AJCC 6th or 7th edition (no changes compared to 6th edition), adjuvant radiation therapy, neoadjuvant or adjuvant chemotherapy, date of last contact, vital status, and cause of death. If clinical N0 patients without lymphadenectomy, we designated as clinical N0 for working stage grouping because pathological Nx could not be applied for stage grouping.

### Statistical Analysis

The study population was divided into male and female groups, and the chi-square testing was applied to compare the proportions of each variable between gender groups. We used Kaplan–Meier methods to calculate the actuarial overall (OS) and cancer-specific (CSS) survivals for the two groups, and also analyzed subgroup differences by stage among females. OS was calculated from the date of initial diagnosis, and a censored variable referred to the patients who were still alive at last follow-up. CSS was calculated from the date of initial diagnosis, and the censored variable referred to the patients who were still alive at last follow-up or died of other causes not related to UTUC. The log-rank test was used to assess this statistical difference. Univariate and multivariate Cox regression analyses were applied to test all the possible prognostic factors of OS and CSS. The analyzed results for prognostic factors were presented as hazard ratio (HR) with 95% confidence interval (CI). All *P*-values were two-sided and <0.05 considered to be statistically significant. The statistical analyses were performed by SPSS Statistics 22.0 (IBM Corp., Armonk, NY, United States).

## Results

The characteristics of this population are listed in Table 1. There were more female patients than male patients (57.9 vs. 42.1%) in this population. There were no differences between the two groups in most characteristics examined, including age at diagnosis, tumor location, synchronous status, papillary architecture, N-stage, radiation therapy, and chemotherapy. However, more female patients were diagnosed at early pT-stages (75.1 vs. 65.3%, *P* = 0.016). More female patients also appeared to have right-sided and low-grade UTUC.

**Table 1:** Patient characteristics of the population.

**Characteristics**	**All (%)**	**Male (%)**	**Female (%)**	***P***
Number	506 (100)	213 (42.1)	293 (57.9)	
Age (years)				0.153
Median	67	67	67	
Range	35–92	35–92	35–88	
< 70	295 (58.3)	132 (62.0)	163 (55.6)	
≥70	211 (41.7)	81 (38.0)	130 (44.4)	
Location				0.197
Renal pelvis	292 (57.7)	130 (61.0)	162 (55.3)	
Ureter	214 (42.3)	83 (39.0)	131 (44.7)	
Laterality				0.001
Right	115 (48.5)	39 (36.8)	76 (58.0)	
Left	122 (51.5)	67 (63.2)	55 (42.0)	
Unknown	269	107	162	
Synchronous				0.136
No	478 (94.5)	205 (96.2)	273 (93.2)	
Yes	28 (5.5)	8 (3.8)	20 (6.8)	
Grade				0.002
Low	24 (8.6)	3 (2.5)	21 (13.1)	
High	255 (91.4)	116 (97.5)	139 (86.9)	
Unknown	227	94	133	
Papillary architecture				0.958
No	263 (52.0)	111 (52.1)	152 (51.9)	
Yes	243 (48.0)	102 (47.9)	141 (48.1)	
pT-stage				0.198
T0/Ta/Tis	85 (16.8)	31 (14.5)	54 (18.4)	
T1	170 (33.6)	67 (31.5)	103 (35.2)	
T2	104 (20.5)	41 (19.2)	63 (21.5)	
T3	133 (26.3)	67 (31.5)	66 (22.5)	
T4	14 (2.8)	7 (3.3)	7 (2.4)	
T3–T4				0.016
No	359 (70.9)	139 (65.3)	220 (75.1)	
Yes	147 (29.1)	74 (34.7)	73 (24.9)	
pN-stage				0.220
N0/Nx	482 (95.3)	200 (93.9)	282 (96.2)	
N+	24 (4.7)	13 (6.1)	11 (3.8)	
Stage group				0.152
0a/0is	83 (16.4)	31 (14.5)	52 (17.7)	
I	168 (33.2)	66 (31.0)	102 (34.8)	
II	102 (20.2)	39 (18.3)	63 (21.5)	
III	117 (23.1)	57 (26.8)	60 (20.5)	
IV	36 (7.1)	20 (9.4)	16 (5.5)	
Radiation therapy				0.290
No	498 (98.4)	208 (97.7)	290 (99.0)	
Yes	8 (1.6)	5 (2.3)	3 (1.0)	
Chemotherapy				0.208
No	463 (91.5)	191 (89.7)	272 (92.8)	
Yes	43 (8.5)	22 (10.3)	21 (7.2)	

The median follow-up time for living patients was 6.9 years. The overall actuarial 5-year OS and CSS rates were 69.4 and 84.9%, respectively. In the univariate analysis for OS, we found that gender, age at diagnosis, architecture, T-stage, N-stage, radiation therapy, and chemotherapy were statistically significant prognostic factors (Table 2). The results were similar in the univariate analysis for CSS, excluding age at initial diagnosis (Table 2). In the multivariate analysis, only being female, aged <70 years, and early T-stage remain statistically significant for improved OS and CSS (Table 3).

**Table 2:** Univariate Cox regression analyses for overall and cancer-specific survivals.

	**Overall survival**			**Cancer-specific survival**		
**Variables**	**HR**	**95% CI**	***P***	**HR**	**95% CI**	***P***
**GENDER**						
Female vs. Male	0.66	0.50–0.86	0.003	0.52	0.32–0.82	0.005
**AGE (YEARS)**						
≥70 vs. <70	1.90	1.44–2.51	<0.001	1.33	0.84–2.12	0.226
**LOCATION**						
Ureter vs. Renal pelvis	0.95	0.72–1.25	0.688	0.75	0.46–1.21	0.234
**LATERALITY**						
Left vs. Right	1.32	0.87–2.01	0.189	1.28	0.74–2.20	0.377
**SYNCHRONOUS**						
Yes vs. No	1.54	0.93–2.57	0.097	1.49	0.60–3.68	0.396
**GRADE**						
High vs. Low	2.33	0.86–6.37	0.098	2.45	0.60–10.10	0.214
**PAPILLARY ARCHITECTURE**						
Yes vs. No	0.73	0.55–0.97	0.030	0.56	0.35–0.90	0.018
**T3–T4**						
Yes vs. No	2.85	2.15–3.78	<0.001	6.78	4.14–11.08	<0.001
**NODE POSITIVE**						
Yes vs. No	3.53	2.17–5.76	<0.001	4.19	2.08–8.45	<0.001
**RADIATION THERAPY**						
Yes vs. No	4.08	1.80–9.26	0.001	5.71	2.07–15.8	0.001
**CHEMOTHERAPY**						
Yes vs. No	2.31	1.54–3.47	<0.001	3.88	2.23–6.78	<0.001

*HR, hazard ratio; CI, confidence interval*.

**Table 3:** Multivariate Cox regression analyses for overall and cancer-specific survivals.

	**Overall survival**			**Cancer-specific survival**		
**Variables**	**HR**	**95% CI**	***P***	**HR**	**95% CI**	***P***
**GENDER**						
Female vs. Male	0.69	0.52–0.91	0.009	0.62	0.38–0.99	0.049
**AGE (YEARS)**						
≥70 vs. <70	2.16	1.62–2.89	<0.001	1.64	1.01–2.67	0.047
**LOCATION**						
Ureter vs. Renal pelvis	1.11	0.83–1.49	0.477	1.01	0.61–1.68	0.976
**SYNCHRONOUS**						
Yes vs. No	1.47	0.86–2.50	0.157	1.16	0.44–3.04	0.760
**PAPILLARY ARCHITECTURE**						
Yes vs. No	0.84	0.62–1.14	0.262	0.78	0.46–1.30	0.334
**T3–T4**						
Yes vs. No	2.50	1.81–3.45	<0.001	5.32	3.09–9.17	<0.001
**NODE POSITIVE**						
Yes vs. No	1.51	0.87–2.63	0.145	1.44	0.65–3.16	0.371
**RADIATION THERAPY**						
Yes vs. No	1.60	0.64–3.99	0.310	1.34	0.42–4.22	0.620
**CHEMOTHERAPY**						
Yes vs. No	1.47	0.91–2.38	0.112	1.77	0.92–3.44	0.090

*HR, hazard ratio; CI, confidence interval*.

**Figure 1:**
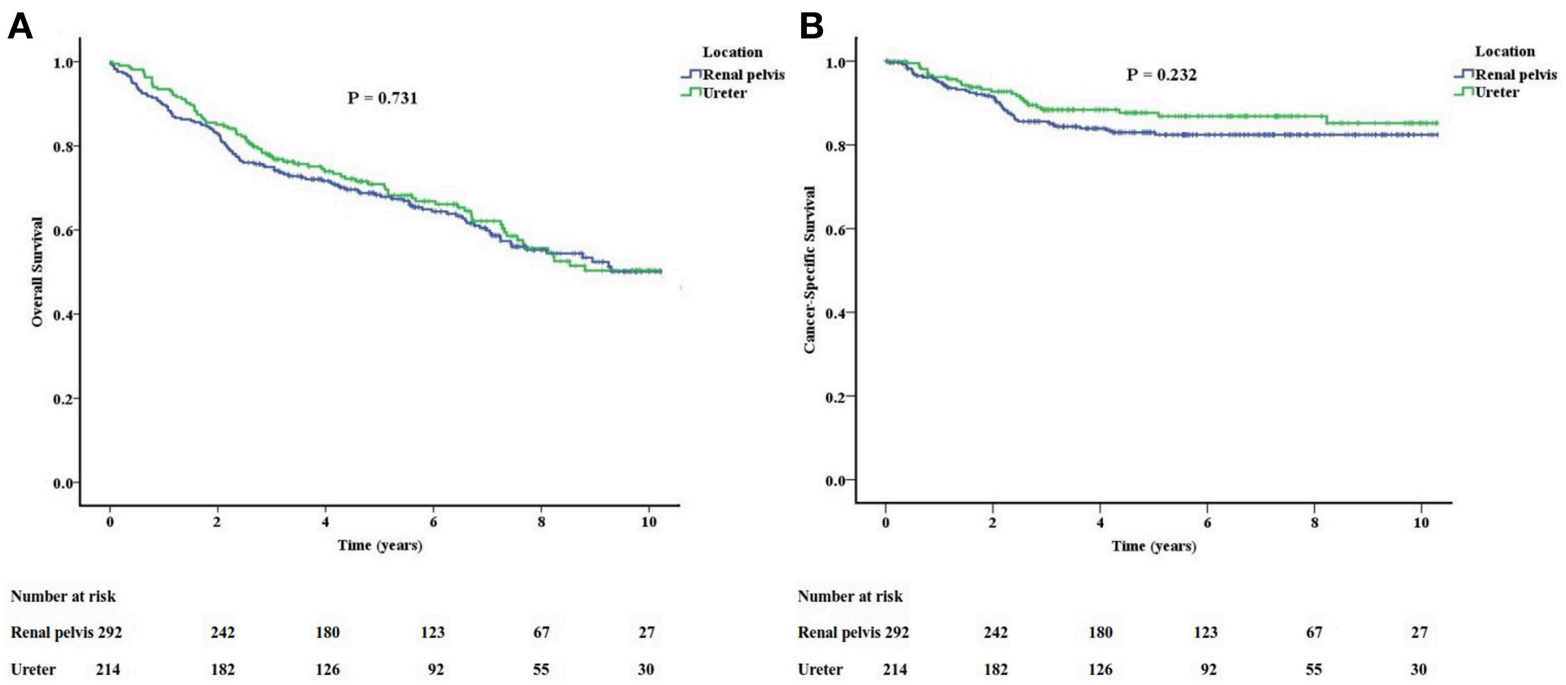
The overall **(A)** and cancer-specific **(B)** survivals between renal pelvis cancer and ureter cancer are not significantly different.

The survival outcomes between renal pelvis cancer and ureter cancer are not statistically different (Figure 1). The actuarial 5-year OS rate was 68.3% in renal pelvis cancer and 70.9% in ureter cancer (*P* = 0.731). The actuarial 5-year CSS rates were 83.0 and 87.7% for renal pelvis and ureter cancers, respectively (*P* = 0.232).

**Figure 2:**
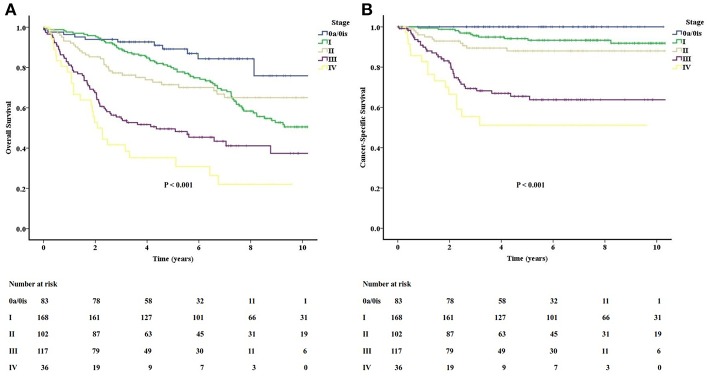
The long-term follow-up of overall **(A)** and cancer-specific **(B)** survivals for different stage groups in the study.

The long-term follow-up of OS and CSS for different stage groups are shown in Figure 2. The actuarial 5-year OS rates of stage 0a/0is, I, II, III, and IV were 89.2, 79.9, 71.5, 49.5, and 35.3%, respectively. The actuarial 5-year CSS rates of stage 0a/0is, I, II, III, and IV were 100, 94.2, 88.0, 65.5, and 51.2%, respectively. The survival curves for CSS were separated well-according to their stage groups. The OS for stages I and II crossed at about the 7-year mark. Besides, both survival curves for OS and CSS were separated obviously between stage II and stage III.

**Figure 3:**
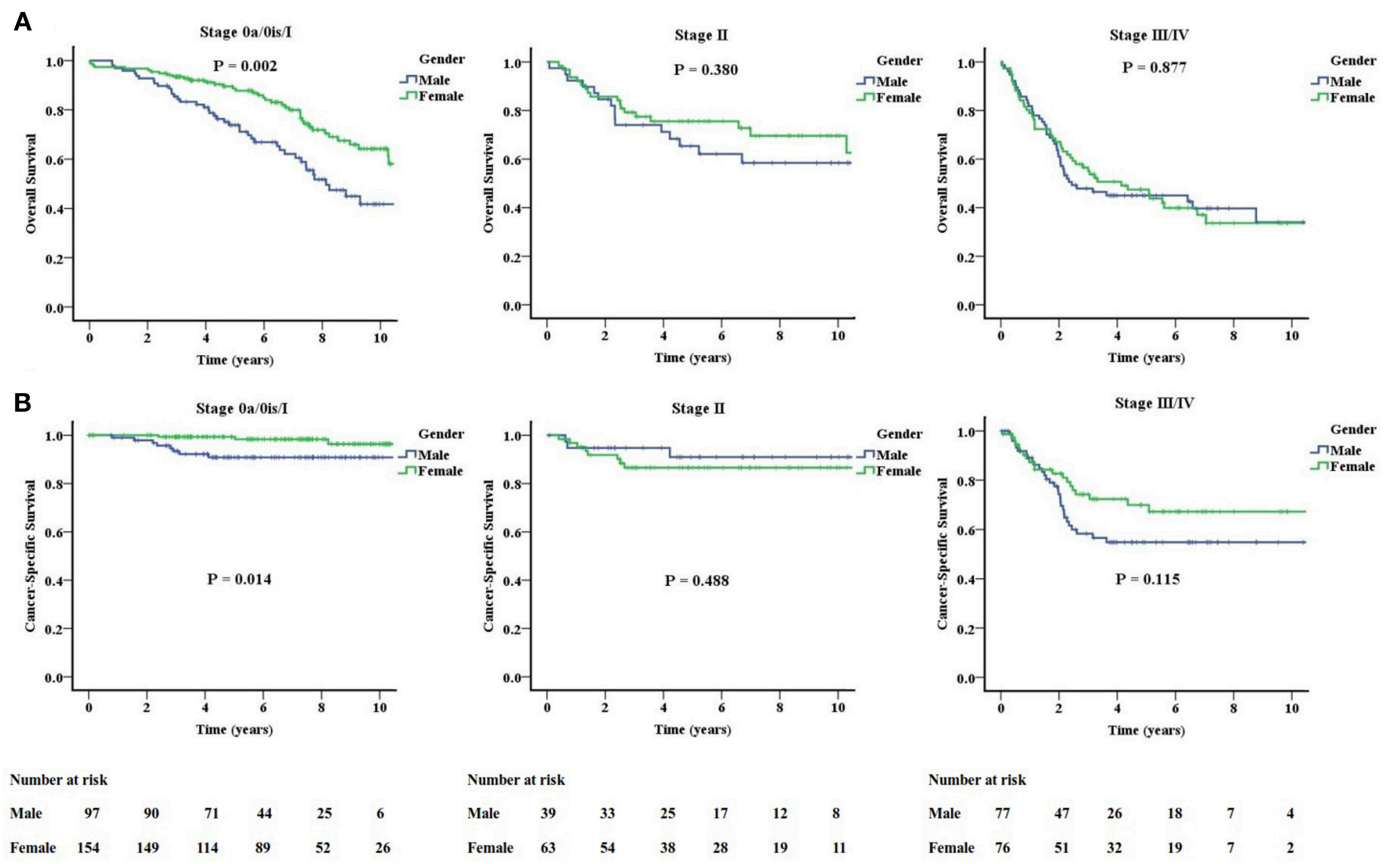
Significantly improved overall **(A)** and cancer-specific **(B)** survivals for females only exist in the stage 0a/0is/I (non-muscle-invasive stage).

Female patients had better OS and CSS than male patients. The actuarial 5-year OS rates were 75.2 and 61.7% for female and male patients, respectively (*P* = 0.003). The actuarial 5-year CSS rates were 89.6 and 78.5% for female and male patients (*P* = 0.005). In the subgroup analysis according to stage 0a/0is/I (non-muscle-invasive), stage II (muscle-invasive), and stage III/IV (non-organ-confined), the gender difference in OS and CSS only persist in the non-muscle-invasive stage (Figure 3). For stage 0a/0is/I, the actuarial 5-year OS rates were 88.7 and 73.8% (*P* = 0.002), and 5-year CSS rates were 99.3 and 90.8% (*P* = 0.014) for female and male patients, respectively. There were no differences for stage II and III/IV patients when gender was examined.

## Discussion

In this study, we found that there were more female patients than male patients in UTUC, and female patients had better survival outcomes than male patients, which confirmed that a strongly different trend for gender distribution and gender-associated outcomes existed for our endemic population as compared with other reports from previous large multicenter series (4–6).

There were also other series which showed more male predominance for UTUC: a population-based study (10) using the Austrian National Cancer Registry database revealed more males than females in UTUC (56.6 vs. 43.4%), which was compatible with the results of large multicenter series (4–6, 11, 12). Another multicenter report from France (13) and small series from Bulgaria (14) found twice as many males as females in UTUC. In Asia, one multicenter report (15) from Japan revealed that the number of males were 2.7 times that of females; in addition, a single institution's study (16) from Korea even reported that the males were nearly quadruple the females. In mainland China, one study from Guangzhou (17) also reported twice as many males as females; however, another patient population (18, 19) in Beijing revealed similar gender distribution such as ours.

Consistent to our report from the same endemic area, several small series (20–23) from Taiwan reported more females than males in UTUC, and the Taiwan Cancer Registry Annual Report in 2015 also revealed that the crude incidence rate was higher in females (9). With our population data which is largest being reported, we can now clearly confirm that the gender distributions in Taiwan (as well as Beijing) are very different from other regions in the world. This observation may have a culturally based explanation: we have a strong custom that most postpartum females consume special nourishment and diets involving herbal medicines daily for at least 1 month after each pregnancy. Consequently, our females have a higher risk of exposure to a potent carcinogen, aristolochic acid, which is present in certain herbal medicines and has been well-known to cause UTUC (8).

There was no significant difference in age distribution between males and females in our study, which was compatible with a previous report (23) from Taiwan; however, several large series (4, 6, 11) reported that their female cases were older than the males. We hypothesized that this may be due to ethnic and environmental differences. Most females in Taiwan may begin to take herbal medicine containing aristolochic acid as early as the beginning of child-bearing age. Age was found to be a significant predictor for survival, which was compatible with several previous reports (6, 11, 24). On the other hand, age was not found to be a significant prognostic factor for OS and CSS in the multivariate analysis from the Beijing series (18).

In this study, the tumor grades in nearly half of the patients were missing because the grading in accordance with WHO classification was not applicable to our cancer registry system, and a number of patients were recorded as unknown as a result. With our limited data, we found that although most patients had high grade tumor, the tumor grade was not a significant prognostic factor, which were compatible with the results of the population in Beijing (18). We also found that significantly more females (13.1%) than males (2.5%) were diagnosed with low grade tumor. As for other pathological factor in our population, tumor architecture in females was not significantly different from those in males, but significantly fewer females were diagnosed with advanced T-stage. However, the exact causes of why more female patients were diagnosed with early T-stage urothelial carcinoma were hard to define. Some studies (6, 10) showed more females diagnosed with advanced T-stage. In contrast, other studies (5, 23) showed fewer females were diagnosed with advanced T-stage, which was compatible with the finding of present study. The reasons for these discrepant results remain poorly understood.

As for gender-associated survival outcome, almost all the previous studies reported that there were no differences between males and females in UTUC (4, 5, 13, 23). One SEER-based study (6) reported that females had a higher cancer-specific mortality rate, but it is not significant in the multivariable competing-risks regression model. The study from Austria (10) ever reported that females had higher overall- and cancer-specific mortality in advanced stages. The study from Guangzhou (17) found that females had worse CSS, and this survival outcome was age-specific and only existed in the patients aged 58 years and higher. To the contrary, a small series from Bulgaria (14) and the patient population in Beijing (18) revealed that females had better OS in their univariate analyses, but it was not statistically significant in the final multivariate analyses.

Our study is first to demonstrate that OS and CSS in females were significantly better than those of males by both univariate and multivariate analyses. Significantly more females than males were diagnosed with low grade tumor and early T-stage might be one of the reasons why female patients had better survival in spite of higher exposure to a potent carcinogen, aristolochic acid. Two clinical studies from Taiwan (20, 25) also reported that bladder recurrence was significantly greater in male patients, and one animal study (26) revealed that both androgens and androgen receptors could promote urothelial carcinoma development and progression. Another clinical study from Taiwan (21) reported that male patients had poor renal outcome which may also be related to poorer cancer outcome in this group.

This gender difference in OS and CSS only exists in the non-muscle-invasive stage and was not present in the muscle-invasive nor non-organ-confined stages. Besides, we found that both survival outcomes obviously decline since stage III, and there were no gender differences in the non-organ-confined stage. We need to intensify the treatment strategy such as offering more adjuvant therapeutic options for both male and female patients in the poorer prognostic groups.

There are some limitations present in our study. In our cancer registry database, some clinical (including performance status and tumor laterality) or pathological variables (such as tumor grade and lymphovascular invasion status) were not fully captured for every patient. The tumor grade, particularly, in the multivariable analyses could bias the results, and we are not sure that the absence of this variable cannot affect the overall results. The cancer registry does not have data regarding patterns of failure or recurrence locally and distantly. Although the data was prospectively collected as an institutional registry, our analyses were still retrospective in nature which may include inherent selection bias. Finally, information including risk factors such as smoking history and herbal medicine use were not included. Surgical extent and operation methods were not recorded in the registry.

## Conclusion

We reported the largest patient population in a single institution and highlighted a characteristically different pattern of gender difference and gender-associated outcome of UTUC after radical nephroureterectomy in an endemic area. We found that females were more common than males in this population, and gender was a significant prognostic factor for OS and CSS. The better survival outcomes for female patients only existed in non-muscle-invasive stage, not in advanced stage disease. Future research should focus on better understanding the epidemiologic risk-factor profile and pathophysiologic differences based on gender and consider intensifying cancer treatments for poorer prognostic groups. The gender-associated outcome of UTUC in Taiwan is distinctly unique as compared to the rest of the world.

## Data Availability

The datasets generated for this study are available on request to the corresponding author.

## Ethics Statement

This study was approved by the Chang Gung Medical Foundation Institutional Review Board (No. 201601404B0).

## Author Contributions

C-CH, Y-TC, H-CH, and C-HL were involved in the conception and design. C-CH, Y-LS, H-LL, TTS, and C-HL were involved in the analysis and interpretation of the data. C-CH, Y-LS, and H-LL drafted the paper. Y-TC, TTS, H-CH, and C-HL revised it critically for intellectual content. All authors gave the final approval of the version to be published.

### Conflict of Interest Statement

TTS is a member of the advisory board for NovoCure Limited, Inc. (New York, NY) which is not related to the production of this work. The remaining authors declare that the research was conducted in the absence of any commercial or financial relationships that could be construed as a potential conflict of interest.
